# Aggression, Social Stress, and the Immune System in Humans and Animal Models

**DOI:** 10.3389/fnbeh.2018.00056

**Published:** 2018-03-22

**Authors:** Aki Takahashi, Meghan E. Flanigan, Bruce S. McEwen, Scott J. Russo

**Affiliations:** ^1^Laboratory of Behavioral Neuroendocrinology, University of Tsukuba, Tsukuba, Japan; ^2^Fishberg Department of Neuroscience and Friedman Brain Institute, Icahn School of Medicine at Mount Sinai, New York, NY, United States; ^3^Laboratory of Neuroendocrinology, The Rockefeller University, New York, NY, United States

**Keywords:** aggression, social stress, immune system, depression, humans, animal models

## Abstract

Social stress can lead to the development of psychological problems ranging from exaggerated anxiety and depression to antisocial and violence-related behaviors. Increasing evidence suggests that the immune system is involved in responses to social stress in adulthood. For example, human studies show that individuals with high aggression traits display heightened inflammatory cytokine levels and dysregulated immune responses such as slower wound healing. Similar findings have been observed in patients with depression, and comorbidity of depression and aggression was correlated with stronger immune dysregulation. Therefore, dysregulation of the immune system may be one of the mediators of social stress that produces aggression and/or depression. Similar to humans, aggressive animals also show increased levels of several proinflammatory cytokines, however, unlike humans these animals are more protected from infectious organisms and have faster wound healing than animals with low aggression. On the other hand, subordinate animals that receive repeated social defeat stress have been shown to develop escalated and dysregulated immune responses such as glucocorticoid insensitivity in monocytes. In this review we synthesize the current evidence in humans, non-human primates, and rodents to show a role for the immune system in responses to social stress leading to psychiatric problems such as aggression or depression. We argue that while depression and aggression represent two fundamentally different behavioral and physiological responses to social stress, it is possible that some overlapped, as well as distinct, pattern of immune signaling may underlie both of them. We also argue the necessity of studying animal models of maladaptive aggression induced by social stress (i.e., social isolation) for understanding neuro-immune mechanism of aggression, which may be relevant to human aggression.

## Introduction: interplay between immune system and central nervous system

The immune system is the body's primary active defense against physical injury and pathogens. These insults cause activation of leukocytes that produce cytokines to promote multiple kinds of inflammatory responses. Cytokines are known to produce an array of sickness behaviors such as reductions in activity, food intake, and social interaction, along with increased sleep and anhedonia (Larson and Dunn, 2001; Dantzer et al., 2008). Psychological stress can trigger cytokine release, and growing evidence has shown an important role for the immune system in regulating negative emotional states as well as personality (Black, 2003; Zalcman and Siegel, 2006; Dantzer et al., 2008; Koolhaas, 2008; Maes et al., 2009; Réus et al., 2015).

To produce deleterious behavioral effects in response to stress, peripheral cytokines must enter and act upon brain circuitry controlling mood and emotion (Menard et al., 2017b). There are two main pathways for peripheral cytokines to affect the central nervous system (CNS): the neural pathway via vagus nerve and the humoral pathway via crossing of the blood brain barrier (for more detail, see review from Hodes et al., 2015; Pfau and Russo, 2015). Within the CNS, activated microglial cells, astrocytes, neurons, and endothelial cells have all been shown to produce several cytokines and express many cytokine receptors (Hopkins and Rothwell, 1995; Allan et al., 2005). Thus, brain cytokines have important roles beyond inflammatory processes and can act as neuromodulators to regulate neuronal transmission and plasticity. For example, one of the major proinflammatory cytokines, interleukin-1β (IL-1β), has been shown to increase metabolism of norepinephrine and serotonin (5-HT) (Dunn, 1992; Linthorst et al., 1994, 1995; Zalcman et al., 1994; Brebner et al., 2000; Anisman et al., 2008). In addition, IL-1β increases the production of corticotrophin-releasing factor (CRF) from the hypothalamus and therefore activates the hypothalamus-pituitary-adrenal (HPA) stress axis (Berkenbosch et al., 1987; Linthorst et al., 1994, 1995; Angeli et al., 1999). IL-1 activates the nuclear factor kappa B (NF-κB) signaling pathway (Osborn et al., 1989), which is well-established to regulate synaptic plasticity (Schneider et al., 1998; Russo et al., 2009; Boersma et al., 2011; Christoffel et al., 2011).

The immune system can also be modulated by the CNS via top-down mechanisms involving nervous and endocrine systems. For example, stress or negative emotions such as anger activates both the HPA axis and the sympathetic-adrenal-medullary (SAM) axis to induce the release of pituitary and adrenal hormones such as adrenocorticotropic hormone, glucocorticoids, prolactin, growth hormone, noradrenaline, and adrenaline. These hormones directly modulate the activity of many immune cells, which express a variety of hormone receptors (for review see Glaser and Kiecolt-Glaser, 2005). For example, glucocorticoids strongly suppress immune cells, and they are widely used in the treatment of inflammatory and autoimmune diseases (Boumpas et al., 1993). Glucocorticoids inhibit the production of pro-inflammatory cytokines by acting directly on glucocorticoid receptors on leukocytes (Lew et al., 1988; Angeli et al., 1999; Dhabhar and McEwen, 1999) (Box 1). The sympathetic nervous system, which regulates physical responses to fight/flight situations, also innervates the hematopoietic stem cell niche located within lymphoid organs and bone marrow to modulate leukocyte differentiation and release through a β-adrenergic receptor mechanism (Elenkov et al., 2000; Bierhaus et al., 2003; Tan et al., 2007) (Box 1).

Box 1 **Biphasic effects of stress on immune response.**

Experiences leading to activation of the autonomic nervous system and HPA axis, as well as other mediators, have biphasic effects on immune responses. One example of this is seen with delayed type hypersensitivity (DTH), an antigen-specific cell-mediated immune response, where acute stress enhances the DTH response while chronic stress has an immunosuppressive effect on the DTH response (Dhabhar and McEwen, 1999). Immune cells “move to their battle stations” and traffic via the circulation to places in the body where they can fight an infection or heal a wound (Dhabhar et al., 2012). Besides adrenaline and cortisol as mediators of this trafficking, IFN-γ, and chemokines are involved (Dhabhar et al., 2000, 2010). IFN-γ plays a role in short-term stress induced immune-enhancement of cell-mediated immunity (Dhabhar et al., 2000), the primary acquisition of immune memory (Dhabhar and Viswanathan, 2005), and in anti-tumor immunity (Dhabhar et al., 2010). However, the actions of IFN-γ may largely come into play after leukocytes have been trafficked to potential sites of immune activation by corticosterone, epinephrine, and norepinephrine (Dhabhar et al., 2012). Parasympathetic and sympathetic activation have synergistic and somewhat opposing effects on immune activation; while activation of the sympathetic response increases inflammatory cytokine production, activation of the parasympathetic response has anti-inflammatory effects (Borovikova et al., 2000; Bierhaus et al., 2003; Matteoli et al., 2014).

## Aggression and the immune system in humans

Aggressive behavior in humans is complex in its expression (i.e., physical and verbal) as well as its causes (i.e., provoked emotion) and consequences on victims (i.e., trauma induced psychopathology) (Box 2). Several mental disorders, such as schizophrenia, psychosis, antisocial personality disorder, impulse control disorder, depression, attention deficit disorder, and autism spectrum disorders accompany escalated forms of aggression toward others (violence) or one's self (self-mutilation) (Connor et al., 2002; Raine et al., 2002; Volavka et al., 2005; Comai et al., 2012; Matson and Jang, 2014; Das et al., 2016).

Box 2 **Aggression study in human and animal models.**

**Human**

Human aggression can be largely categorized into reactive aggression (impulsive and hostile) and premediated (instrumental) aggression. Quantification of aggression in human is often conducted by psychometric inventories. Buss-Durkee Hostility Inventory (BDHI, Buss and Durkee, 1957) or its modified Buss and Perry Aggression Questionnaire (BPAQ, Buss and Perry, 1992) contains subscales for hostility (cognitive aspect of aggression including feelings of ill-will and injustice), anger (affective aspect of aggression including physiological arousal and preparation for aggression), and physical and verbal aggression (behavioral aspect of aggression including instrumental or motor components of behavior). The Cook-Medley Hostility (Ho) Scale is another commonly used questionnaire (Cook and Medley, 1954) that measures cognitive (cynicism and hostile attribute), affective (hostile affect), and behavioral (aggressive responding) aspects of aggression. Other studies use methods to provoke anger in experimental settings and quantify participants' aggressive responses toward fictitious competitors (for review, see Miczek et al., 2002).

**Animal models**

Aggressive behavior in animals is species-typical behavior and it differs depending upon the social system of the species, and includes factors such as territorial aggression, dominance-related aggression, and maternal aggression (Miczek and Fish, 2006). Moreover, aggressive behavior in animals has been traditionally separated into offensive vs. defensive forms of aggression. Offensive aggression is motived by resource control and threat to those resources, whereas defensive aggression is motived by danger of harm to the individual itself (Blanchard and Blanchard, 2006). In the rodent model, aggressive behavior is often quantified by using the resident-intruder test. This test is conducted in the home-cage of a resident male (or sometimes resident female or dam) where an intruder male is introduced. Latency to first attack behavior (often a bite) is measured and used as an index of readiness to initiate aggressive behavior. Animals with shorter attack latency are considered to have higher aggression. In addition, the overall frequency and duration of aggressive acts are measured. Importantly, while dominance is a trait often associated aggression, for the purpose of this review it is important to highlight some distinctions. Dominance-related aggression typically occurs more often in species with defined social hierarchies, such as non-human primates, and is measured by ethological observation to record animals' interactive behaviors with other members in their habitat (either captivity or the field). Importantly, social dominance does not simply reflect trait aggressiveness (Buwalda et al., 2017) but can result from other factors such as ability to mobilize support or anxiety in monkeys and rats (de Waal, 1998; van der Kooij et al., 2017).

Increasing evidence shows that there is an important relationship between aggression traits and the immune system. For example, it has been reported that immunotherapy to treat patients with hepatitis C by chronic administration of interferon alpha (IFN-α) increases irritability and anger/hostility in some patients (McHutchison et al., 1998; Kraus et al., 2003; Lotrich et al., 2013). Also, hostile marital relationships are associated with slower wound healing and dysregulated cytokine production at wound sites (Kiecolt-Glaser et al., 2005). Pathological levels of aggression in intermittent explosive disorder (IED) or psychosis appear to be linked to heightened proinflammatory cytokines (Coccaro et al., 2014; Das et al., 2016). A similar relationship is observed in healthy individuals with high aggression as a personality trait. As summarized in Table 1, these individuals consistently showed higher circulating cytokines such as interleukin-6 (IL-6) and tumor necrosis factor-α (TNF-α), as well as C-reactive protein (CRP) than non-aggressive individuals (Box 3). However, these correlations varied depending on the subtype of aggression scale used. In one recent study, it was shown that the behavioral, but not cognitive nor affective, subscale of hostility positively correlated with IL-6 and CRP (Marsland et al., 2008). Therefore, elevations in basal levels of circulating proinflammatory cytokines, i.e., IL-6 or CRP, appear to be related to the behavioral aspect of aggression traits. Whether this relationship is direct or indirect must be addressed using animal models.

**Table 1:** Relationship between aggression trait or state and immune responses in human studies.

**Condition**	**Subjects**	***N***	**Age**	**Sample**	**Stimulation**	Scale^*^	**Cytokines**	Relationship^**^	**Reference**
Basal	Intermittent Explosive Disorder (IED) males and females	197	32–35.8	Plasma	–	LHA	CRP	+ (*r* = 0.41, *p* < 0.001)	Coccaro et al., 2014
							IL-6	+ (*r* = 0.36, *p* < 0.001)	
Basal	Healthy males and females	855	20–54	Plasma	–	Ho, BPAQ (Behavioral hostility)	IL-6	+ (β = 0.09, *p* = 0.04)	Marsland et al., 2008
							CRP	+ (β = 0.08, *p* = 0.04)	
Basal	Healthy males and females	6814	45–84	Serum	–	Ho	CRP	+	Ranjit et al., 2007
							IL-6	+	
							fibrinogen	+	
Basal	Older males and females (caregiver)	214	69.1	Plasma	–	Ho	CRP	+ (β = 0.18, *p* < 0.05)	Graham et al., 2006
							IL-6	No	
Basal	Healthy non-smoking males	62	18–50	Monocyte	LPS	BPAQ	TNF-α	+ (*r* = 0.35, *p* = 0.007)	Suarez et al., 2002
Basal	Healthy females	44	23–49	Monocyte	LPS	Ho	IL-1α	+ (β = 0.03, *p* = 0.02)	Suarez et al., 2004
							IL-1β	+ (β = 0.02, *p* = 0.06)	
							IL-8	+ (β = 0.05, *p* = 0.01)	
Basal	Healthy males (with military records)	304	30.7	T cells, B cells, and NK-cells	T-cell mitogen	Ho	IL-6, MCP-1 etc	**–** (β = −0.29), *p* < 0.001	Mommersteeg et al., 2008
							IL-4, IL-5, IL-10	+ (β = 0.20, *p* < 0.01)	
							IL-2, TNF-α, IFN-γ	+ (β = 0.24, *p* < 0.001)	
Basal	Healthy males (with military records)	4415	30–48	Whole blood	–	Aggressive behavior scale (DSM-III antisocial personality disorder)	T cell (CD4) number	+ (β = 0.17, *p* < 0.001)	Granger et al., 2000
							T cell (CD8) number	No	
							B cell number	+ (β = 0.20, *p* < 0.001)	
Basal	Healthy males and females	38	32.5	CSF	–	Composite aggression score (LHA + BDHI)	IL-6	No	Coccaro et al., 2015
							Soluble IL-1 receptor II	+ (*r* = 0.35, *p* = 0.03)	
State (2 h before rugby match)	Male rugby athletes	20	27.2	Serum	–	State-Trait Anger expression Inventory-2	IL-1β	+ (β = 0.60–0.72, *p* < 0.05)	Pesce et al., 2013
State (Conflict/social support session)	Healthy marital couples	42	22–77	Plasma	–	The Rapid Marital Interaction Coding System	IL-6	+	Kiecolt-Glaser et al., 2005
							TNF-α	+	
State (Anger recall interview)	Healthy non-smoking males	58	18–65	Plasma	LPS	Total negative score	IL-1β	No	Suarez et al., 2006
							IL-6	+ (males with high insulin resistance)	
							TNF-α	**–** (males with low insulin resistance)	
State (Stressful event self-discloser)	Male college undergraduate students	43	20	NK cell activity	–	Ho	NK cytotoxicity	+ (males with high hostility)	Christensen et al., 1996
State (Discussion of marital problem)	Married couples	41	30.9–31.9	NK cell activity	–	Ho	NK cytotoxicity	+ (*r* = 0.71, *p* < 0.01 in high hostile males) **–** (*r* = −0.63, *p* < 0.01 in low hostile males)	Miller et al., 1999

* LHA, life history of aggression; Ho, Cook-Medley Hostility Sale; BPAQ, Buss and Perry Aggression Questionnaire; BDHI, Buss-Durkee Hostility Inventory. Total negative score includes "anxious, depressed, upset, tense, angry, frustrated, agitated, nervous, irritated, and sad.”

** *Significant relationship [either positive (+) or negative (–) direction] between aggression scores and cytokines. r, correlation coefficients; β, beta coefficients from the regression analysis; B, unstandardized regression coefficient from the Structural equation modeling (SEM). No statistic values were indicated if the study used other types of analysis*.

Box 3 **IL-1, IL-6, CRP, TNF-α.**

IL-1 (interleukin-1) is a potent pro-inflammatory cytokine first identified as an endogenous pyrogen due to its ability to affect the hypothalamic thermoregulatory center. Currently, there are 11 cytokines in the IL-1 super family (for review, see Allan et al., 2005). Two major subtypes of IL-1 ligands, IL-1α and IL-1β, bind to IL-1 receptors (IL-1R) to activate intracellular cascades such as NF-κB and mitogen-activated protein kinases (MAPKs), and trigger the transcription of multiple inflammation-associated genes including IL-6 and TNF-α. There is also a ligand known as IL-1RA that antagonizes IL-1R to inhibit downstream signaling. Many types of cells in both the peripheral and central immune system produce IL-1 and express IL-1 receptors, including leukocytes, endothelial cells, adipocytes, fibroblasts, neurons, and glial cells.

IL-6 (interleukin-6) is a cytokine that can exhibit either anti-inflammatory or pro-inflammatory properties depending on whether the IL-6 receptor and glycoprotein 130 (gp130) signal transducer are soluble or membrane bound. As is the case with IL-1, IL-6 is produced in many cell types. It was originally identified as B-cell differentiation factor, but it also has a variety of additional functions outside of B cells such as production of acute-phase proteins from liver, angiogenesis, T-cell differentiation, bone metabolism, and neuronal growth (for review, see Hodes et al., 2016).

CRP (C-reactive protein) is one of the acute-phase proteins from the liver activated in by pro-inflammatory cytokines as early a response to inflammation. CRP acts as a pattern recognition molecule that binds to the surface of several microbes and dead cells, and it has been used as a sensitive but non-specific marker of inflammation and infection (Pepys and Hirschfield, 2003).

TNF-α (tumor necrosis factor alpha) is a pro-inflammatory cytokine that was originally identified as a cytotoxic factor produced by lymphocytes and macrophages. More recently TNF-α has been shown to trigger the induction of an array of pro-inflammatory cytokines to regulate cell proliferation, differentiation, and cell death (Aggarwal et al., 2012).

High states of anger (acute episodes of anger) also induce proinflammatory cytokine release. Marital couples show increases in plasma IL-6 and TNF-α after conflict interactions compared to after supportive interactions, and these increases in cytokines were larger in couples who showed higher hostile behaviors during their conflict interactions (Kiecolt-Glaser et al., 2005). Interestingly, the expectation of an aggressive encounter can also increase circulating proinflamatory cytokine levels as well. Rugby athletes displayed increased IL-1β levels in their blood 2 h before a match, when state anger is high, compared to basal levels (Pesce et al., 2013). Furthermore, IL-1β levels 2 h before the match were positively correlated to anger score. Thus, both an aggressive experience and an expectation of an aggressive event are accompanied by state-related increases in inflammatory cytokines. It is possible that the readiness to provoke anger and aggressive behavior depends on these individual differences in cytokine production. But also, since noradrenaline triggers activation of monocytes to produce inflammatory cytokines (Bierhaus et al., 2003), sympathetic activation by aggressive events or their expectation might be the cause of this increased cytokine production.

Immune responses are largely divided into two categories: a rapid general immune response (innate immunity) and an acquired delayed immune response (adaptive immunity). Lipopolysaccharide (LPS) is an endotoxin that composes the outer surface membrane of gram-negative bacteria, acts as a pathogen-associated molecular pattern (PAMPs), and stimulates the innate immune response. Studies examining the role of the innate immune response on aggression show that there is a positive correlation between LPS-stimulated monocyte TNF-α expression and aggression (in hostility and behavioral, but not anger, subscales) in healthy males (Suarez et al., 2002). Similarly, monocytes isolated from females with high hostility released more IL-1 and IL-8 than those isolated from low hostility females after LPS-stimulation (Suarez et al., 2004). There have also been reports of increased natural killer (NK) cell cytotoxicity in highly hostile individuals (Christensen et al., 1996; Miller et al., 1999). Thus, individuals with high aggression traits tend to have high innate immune responses, though it is still unclear whether these are causally linked to aggression.

Only a few studies have examined the adaptive immune system in anger/hostility. Of note, one study found that there was a significant positive correlation between the frequency of T and B lymphocyte numbers and past aggressive acts; however, this relationship was only clear in individuals with moderate aggression, but not in highly aggressive individuals (Granger et al., 2000). Another study found that hostility is positively correlated with the release of both pro-inflammatory (TNF-α and IL-2) and anti-inflammatory (IL-4 and IL-10) cytokines from isolated T-cells (Mommersteeg et al., 2008). T-cell driven IL-6, however, was negatively correlated with hostility in the aforementioned study, which opposes the results observed in studies where cytokines were measured from whole serum/plasma or directly in monocytes. This discrepancy suggests that the relationship between aggression traits and inflammatory response is different depending on the leukocyte cell types studied.

Studies measuring cytokines from cerebrospinal fluid (CSF) contrast with those measuring them from peripheral blood. There is no observed correlation between IL-6 levels in the CSF and aggression (Coccaro et al., 2015). Instead, there is a positive correlation between levels of soluble IL-1 receptor II (sIL-1R2) in the CSF and aggression history (Coccaro et al., 2015). IL-1R2 and its soluble form sIL-1R2 act as decoy receptors for IL-1 and inhibit IL-1 mediated signal transduction (Allan et al., 2005). The sIL-1R2 binds to IL-1β with high affinity, and thus the level of IL-1R2 was used as an indirect measurement of IL-1β in the CNS in their study.

These studies in humans highlight the correlational relationship between aggression and the immune system. In the later section, we discuss findings from animal models where more causal relationships between the immune system and aggressive behaviors are beginning to be examined.

## Link between aggression, depression, and the immune system in human studies

Similar to the findings from aggression studies, increased circulating IL-6 has been observed in humans suffering from major depression (Maes et al., 1997; Kiecolt-Glaser et al., 2003; Hodes et al., 2014; Kiraly et al., 2017). Thus, increased circulating IL-6 seems to be one of the important endophenotypes in depressive-like behaviors as well. Although aggression (violence) and depression are phenotypically very different behavioral outputs, both aggression and depression are triggered by social stress. In fact, suicidal behavior, the most problematic consequence of depression, can be considered as a form of escalated aggression toward the self, and a high comorbidity of suicide and aggression has been observed in human patients (McCloskey and Ammerman, 2018). Thus, it is possible that aggression and depression share certain biological mechanisms.

Repeated immunotherapy to treat patients with acquired immune deficiency syndrome, autoimmune disease, or hepatitis C increases depression as well as anger/hostility (McHutchison et al., 1998; Kraus et al., 2003). In addition, epidemiological evidence has shown that individuals with psychological traits of either depression or hostility have a greater risk of developing coronary heart disease (CHD), which is a known inflammatory disease (Rozanski et al., 1999; Smith and Ruiz, 2002; Betensky and Contrada, 2010). Both male and female individuals with high hostility displayed higher plasma IL-6 levels than non-hostile controls only when they concurrently suffered high depression symptoms (Suarez, 2003). This positive correlation between hostility and IL-6 levels was absent among individuals with low depression symptoms. A similar pattern was observed in another study, where the authors found a significant interaction between hostility and depression symptoms for serum IL-6 and CRP (Stewart et al., 2008). A much larger study of both males and females confirmed a positive correlation between hostility and CRP in highly depressed individuals, but also found no relationship between these measures in people with low levels of depression (Brummett et al., 2010). In contrast, other studies observed a positive correlation between hostility and IL-6 or TNF-α only in individuals with low levels of depression (Miller et al., 2003). This study reported that all individuals with high levels of depression showed high levels of IL-6 regardless of hostility, and thus the relationship between hostility and circulating cytokine levels was not observed. In other experimental designs, the relationship between hostility and cytokines remained even after correcting for depression phenotypes (Marsland et al., 2008). Given the inconsistent results from these correlational studies in humans, far more work is needed to elucidate whether disruptions within the immune system are a common endophenotype for both depression and anger/hostility traits in humans.

## Animal models of aggression and the immune system

In contrast to human aggression research, which mainly focuses on examining anger and hostility as negative emotional states with pathological aspects that could be the matter of clinical concern, aggression research in animals must consider its ethological and evolutionary importance. Aggression has an adaptive significance for most animal species and is critical for acquiring and protecting territory, food, reproductive mates, and offspring. In animals with hierarchical societies, aggressive behavior is thought to help individuals gain and maintain higher social status (Box 2). It has been shown that aggressive behavior, especially the experience of winning, has rewarding properties in animals and repeated aggressive experience may lead to compulsive, pathological aggression that is highly reinforcing (Fish et al., 2002; Falkner et al., 2016; Golden et al., 2016, 2017). Since aggressive behavior poses a strong risk of injury, it is reasonable to assume that animals with high levels of aggression would have stronger immune responses in order to actively recover from injury and to protect themselves from infection.

As with humans, differences in peripheral immune function have been observed between high and low aggressive non-human primates and rodents. For example, baboons with higher hierarchical status within the group showed faster wound healing than subordinate individuals in the wild (Archie et al., 2012). Male cynomolgus monkeys with the lowest social status had rates of infection by adenovirus five times greater than monkeys with higher social rank (Cohen et al., 1997). Also, more aggressive cynomolgus monkeys had higher lymphocyte numbers than less aggressive monkeys when they were infected by herpes B virus (Line et al., 1996). In mice, female BALB/c mice with high aggressive behavior were less vulnerable to tumor induction by murine sarcoma virus than low aggression BALB/c females (Amkraut and Solomon, 1972). In agreement with this, highly aggressive C57BL/6 and CBA male mice show stronger immune response (increase in plaque- and rosette-forming cell numbers) toward immunization with protein antigen than submissive males (Devoino et al., 1993). There was also a positive correlation between aggression traits and experimental autoimmune encephalomyelitis (EAE) response in a wild rat population whereby aggressive male rats were more susceptible to experimentally-induced autoimmune disease (Kavelaars et al., 1999), suggesting that aggressive individuals have highly activated immune systems (Table 2). These data are in some conflict with data obtained from humans, as highly aggressive humans tend to have higher concentrations of proinflammatory cytokines and slower wound healing than less aggressive humans (Kiecolt-Glaser et al., 2005). It is possible that activation of the immune system is adaptive in aggressive or dominant individuals but can become maladaptive in extreme cases of pathological aggression, which make up most of the cases in human studies. This hypothesis needs further testing.

**Table 2:** Relationship between aggression and peripheral immune responses in rodent models.

**Condition**	**Animal model**	**Strain**	**Sex**	**Stimulation**	**Sample**	**Cytokines etc**	**Effect**	**Reference**
Basal	Aggression selection line	NC900 and NC100 mouse (Originated from ICR)	Male	3-methylcholanthrene	Animal	Number of animals with tumors	NC900 < NC100	Petitto et al., 1993
					Splenocytes	NK cytotoxicity	NC900 > NC100	
Basal	Aggression selection line	NC900 and NC100 mouse (Originated from ICR)	Male	T cell mitogen concanavalin A (ConA)	Splenocytes	T cell proliferation	NC900 > NC100	Petitto et al., 1994
					Splenocytes	IL-2	NC900 > NC100	
					Splenocytes	IFN-γ	NC900 > NC100	
				LPS	Splenocytes	B cell proliferation	–	
Basal	Spontaneous fighting female and non-fighting female	BALB/c mouse	Female	Murine sarcoma virus	Animal	Tumor size	Fighter < Non fighter	Amkraut and Solomon, 1972
Basal	Resident-intruder test	Wildtype rat	Male	Experimental autoimmune encephalomyelitis (EAE)	Animal	Disease score	Short attack latency > Long attack latency	Kavelaars et al., 1999
3 days confrontations	Aggressive and submissive males in resident-intruder test	C57BL/6 mouse	Male	B16F10 melanoma injection	Animal	Number of metastasis in the lung	Aggressive < Submissive	Sá-Rocha et al., 2006
				*Staphylococcus aureus*	Blood neutrophils	Basal oxidative burst	Aggressive > Submissive	
					Blood monocytes	Basal oxidative burst	Aggressive > Submissive	
				–	Blood lymphocytes	NK cell cytotoxicity	Aggressive > Submissive	
				–	Spleen lymphocytes	NK cell cytotoxicity	Aggressive > Submissive	
10 days confrontations	Sensory contact	CBA/Lac mouse	Male	Immunization with Sheep red blood cells	Splenocytes	Plaque forming cells	Aggressive > control = Submissive	Devoino et al., 1993
						Rosette-forming cells	Aggressive > control = Submissive	
		C57BL/6J mouse				Plaque forming cells	Aggressive = control > Submissive	
						Rosette-forming cells	Aggressive = control > Submissive	
10 and 20 days confrontations	Sensory contact	C57B/6J mouse	Male	–	Splenocytes	IL-2	20 day > 10 day aggression > control	Idova et al., 2016
						TNF-α	–	
				ConA		IL-2	20 day = 10 day aggression > control	
				LPS		TNF-α	20 day = 10 day aggression < control	
20 days confrontations	Aggressive and submissive males in 15 min confrontations	C57BL/6J mouse	Male	–	Serum	IL-1a	–	Stewart et al., 2015
						IL-2	–	
						IL-4	–	
						IL-6	Aggressive = control (<) Submissive	
						IL-7	Aggressive = control < Submissive	
						IL-10	Aggressive > control = Submissive	
						IL-15	Aggressive = control < Submissive	
						MCP-1	–	
						VEGF	Aggressive = control < Submissive	

Individual differences in peripheral immune responses are also reported in forward genetic models of aggression in which animals are selectively bred for aggressive behavior. High aggression NC900 mice and low aggression NC100 mice have been selected over generations from the ICR outbred founder population (Cairns et al., 1983; Gariepy et al., 1996). Interestingly, the NC900 line showed reduced vulnerability to tumor development after calcinogen treatment than the low aggression NC100 line (Petitto et al., 1993). Furthermore, splenic NK cytotoxic activity was also higher in NC900 mice, and exposure to a T cell mitogen caused greater splenic T cell proliferation and increased production of proinflammatory cytokines IL-2 and intereferon-gamma (IFN-γ) in NC900 mice (Petitto et al., 1993, 1994). Thus, aggressive NC900 mice have stronger NK cell and T cell immune responses than non-aggressive NC100 mice. These differences were observed without having any aggressive experience, suggesting that they may reflect trait-like immunity differences.

Studies in constitutive gene knockout mice support the involvement of the immune system in aggressive behaviors. Deletion of TNF receptors, TNF-R1 and TNF-R2, reduced the duration of aggressive behaviors in the resident-intruder test in male mice (Patel et al., 2010). This is in line with findings from human studies in which TNF-α is increased in highly aggressive individuals (Table 1). On the other hand, IL-6 knockout mice showed shorter attack latency and increased frequency of aggressive behaviors in the resident-intruder test (Alleva et al., 1998). This same study showed that overexpression of IL-6 had no effect on inter-male aggression, but increased non-agonistic social interaction behaviors such as anogenital sniffing. Although interpretation of the results from these knockout mice is complex because of possible compensatory changes in other cytokines throughout the developmental period, these results strongly indicate functional involvement of the immune system in aggressive behaviors.

Although there are relatively few studies that have investigated the role of brain cytokine signaling in aggression, a handful of studies performed in a cat defensive rage model suggest that it functionally promotes defensive aggression (Zalcman and Siegel, 2006). In this model, electrical activation of either the periaqueductal gray (PAG) or medial preoptic area/hypothalamus causes cats to express a range of defensive aggressive behaviors in response to threat such as hissing, pupillary dilatation, retraction of the ear, as well as increases in blood pressure and heart rate (Siegel et al., 1999). It has been shown that IL-1β, IL-2, and their receptors are localized in a variety brain regions including the PAG and the medial hypothalamus (Bhatt et al., 2005; Hassanain et al., 2005). The local administration of IL-1β into the medial hypothalamus caused an enhancement of the defensive rage response (reduction of attack latency) after PAG stimulation (Hassanain et al., 2003). This pro-aggressive effect of IL-1β injection into the medial hypothalamus was blocked by the 5-HT_2_ receptor antagonist LY-53857. Importantly, strong co-localization of 5-HT_2C_ receptors and IL-1 type 1 receptors (IL-1RI) in the medial hypothalamus may reflect the fact that IL-1β and 5-HT are activating the same population of neurons in this region to enhance defensive rage responses (Hassanain et al., 2005). In contrast to IL-1β, microinjection of IL-2 into the medial hypothalamus suppressed defensive rage behavior (Bhatt et al., 2005). The suppressive effect of IL-2 was blocked by pretreatment with a GABA_A_ receptor antagonist into the medial hypothalamus, suggesting that the effect of IL-2 in the medial hypothalamus is mediated through GABA_A_ receptors (Bhatt et al., 2005). However, IL-2 also facilitated defensive rage behavior when it was microinjected into the PAG (Bhatt and Siegel, 2006), and thus the function of cytokines for defensive rage depends heavily on the brain area in which it is expressed. Also, the function of cytokines might be different depending upon the type of aggression. In mouse territorial aggression models using a resident-intruder test, it has been shown that systemic injection of IL-1β causes a strong increase in attack latency concomitant with a reduction in the total duration of aggressive behaviors (Cirulli et al., 1998), indicating that systemic IL-1β has a suppressive effect on intermale offensive aggression. However, this study used only systemic treatment of IL-1β and its effect in the brain has to be studied in offensive aggression.

In summary, both peripheral cytokines and cytokines in the brain have important modulatory roles in both offensive and defensive aggression. Further studies to examine the complex neural circuitry in which cytokines act to affect aggressive behavior will be necessary to understand the extent of neuro-immune interactions in aggression.

## Animal model of depression and the immune system

A large body of evidence shows that both chronic and repeated exposure to social defeat stress, which leads to depression-like behaviors in stress susceptible individuals, has a significant effect on the immune system. Currently, we understand far more about the detailed biological mechanisms underlying the link between social defeat stress and immune activation than we do between aggression and immune activation (for reviews, see Hodes et al., 2015; Pfau and Russo, 2015; Ménard et al., 2017a; Weber et al., 2017). Chronic social defeat stress has been used as an animal model of depression with high ethological and face validity (Miczek et al., 2008; Golden et al., 2011). Disruption of established social hierarchy in the home cage by repeated intrusions of a large dominant male has been shown to cause intensive stress and increases circulating corticosterone in male C57BL/6 mice (Avitsur et al., 2001, 2002). Despite the known immuno-suppressive effect of corticosterone, animals who underwent this social disruption procedure displayed an increased number of splenic monocytes and elevations in IL-6, IL-1β, and TNF-α release after endotoxin LPS stimulation compared to unstressed controls (Stark et al., 2001; Avitsur et al., 2003, 2005; Bailey et al., 2009). Interestingly, these studies also found that social stress caused glucocorticoid receptor (GR) desensitization in splenic monocytes, making them insensitive to inhibition by glucocorticoids and further exacerbating the pro-inflammatory effects of stress (Stark et al., 2001; Avitsur et al., 2002; Jung et al., 2015). Furthermore, monocytes expressing GR lost the ability to efficiently translocate GR into the nucleus, and thus were unable to suppress NF-κB activity (Quan et al., 2003). These changes in the properties of spleen monocytes in socially disrupted animals were mediated by increased norepinephrine and epinephrine release in the blood and spleen (Hanke et al., 2012). These results suggest that repeated social stress results in abnormal activation of the immune system through a loss of negative feedback signaling via corticosterone.

The peripheral immune system has also been implicated in determining individual vulnerability to social stress. For example, Hodes et al. (2014) examined the levels of circulating cytokines in susceptible and resilient C57BL/6 male mice after 10 days of repeated social defeat stress. Susceptible animals that developed social avoidance after repeated defeat experiences, exhibited higher IL-6 in their serum compared to stress-resilient mice as well as non-defeated control mice. Transplantation of bone marrow from susceptible donor males into host control males caused an increased social aversion following acute social stress, indicating that leukocytes are at least partly responsible for stress susceptibility. Interestingly, there was a preexisting difference in leukocytes between susceptible and resilient males such that susceptible animals had more circulating leukocytes and produced more IL-6 after LPS stimulation than resilient animals (Hodes et al., 2014). Increased peripheral IL-6 levels were also observed in susceptible female mice in a newly developed female social defeat stress model, indicating that IL-6 is a common mechanism mediating social stress susceptibility among the sexes (Takahashi et al., 2017). In addition to exhibiting differences in the immune response to stress, we also show that permeability of the blood brain barrier is different between resilient and susceptible male mice (Menard et al., 2017b). Stress susceptible mice display damaged and leaky blood vessels in the nucleus accumbens (NAc) due to loss of Claudin 5 expression, a molecule that helps form tight junctions between endothelial cells that make up the blood brain barrier (BBB). This impairment in BBB permeability allows more blood IL-6 to enter the brain and promotes depression-like behaviors following social defeat. Future work is needed to understand at the molecular level how social stress primes the neuro-immune axis to open the BBB and promotes a permissive environment for immune-CNS interactions.

## Biological mechanisms linking aggression, depression, and immune function

Broadly, the studies described above support the notion that both aggressive and socially-defeated animals display increased production of peripheral cytokines. However, mounting evidence suggests that this simple view does not capture the complex dynamics of cytokine levels in socially stressed animals. For example, repeated aggressive encounters over 20 days caused increases of serum proinflammatory cytokines IL-6, IL-7, and IL-15 in loser, but not winner, male C57BL/7 mice (Stewart et al., 2015). Importantly, there was an increase in the anti-inflammatory cytokine IL-10 only in winner males. Further studies are required to understand whether these changes in cytokines observed in winners and losers represent common responses to social stress, or if there are specific immune responses that differ between winners and losers (such as specific activation of anti-inflammatory IL-10 in winners) that function to alter subsequent behavioral outputs. One hypothesis is that dominant individuals have well-balanced activation of both pro-inflammatory and anti-inflammatory cytokines to actively cope with injury or infections caused by aggressive interactions. In contrast, the experience of repeated social defeat stress, which causes a depression-like phenotype, may induce dysregulation of the immune system leading to a pro-inflammatory state of the animal.

The stress hormone corticosterone increases in both dominant and subordinate animals during an aggressive encounter (Covington and Miczek, 2005), but only subordinate animals show over-activation of HPA axis and long-term hypercortisolism after continuous subordination (Ely and Henry, 1978; Sapolsky, 1989). For example, dominant baboons showed normal activation of cortisol secretion by corticosterone-releasing factor (CRF) and suppression by glucocorticoid negative feedback. By contrast, continuously subordinated baboons displayed hypercortisolism, a blunted response to CRF, and resistance to glucocorticoid-induced negative immune feedback (Sapolsky, 1990). Similarly, in human air traffic controllers with high competence and satisfaction showed a positive correlation between plasma cortisol and amount of workload (number of airplanes under their control), but individuals with low competence showed blunted or disregulated cortisol response (Rose et al., 1982). However, recent work suggests that both the stability of the hierarchy as well as the species under investigation influences findings of whether subordinate status is associated with the highest rate of physical and psychological stressors (Abbott et al., 2003). The immune system also responds to agonistic encounters (acute social stress) in both aggressive dominant and submissive defeated individuals. However, as we have discussed in former sections, dominant animals mount strong adaptive immune responses that protect them from infection or enhance their recovery from injury, while defeated animals develop more prolonged dysregulation of the immune system that leads to pathological physiology and behavioral phenotypes. While causal data linking corticosterone to immune-mediated aggression is not yet available, it is one of the key mediators of brain-immune interactions (Spencer et al., 1991; Dhabhar and McEwen, 1999; Marques-Deak et al., 2005; also see Box 1) making it an attractive candidate for further study.

5-HT is one of the most well-studied neurotransmitters involved in the pathophysiology of both depression and escalated aggression (Olivier, 2015; Manchia et al., 2017). Serotonin reuptake inhibitors (SSRIs), the most widely used pharmacological treatment for depressed patients, suppress escalated forms of aggression in rodent models (Pinna et al., 2003; Caldwell and Miczek, 2008; Mikics et al., 2017) and owner-directed aggression in dogs (Dodman et al., 1996). It has been shown in a variety of studies that peripheral cytokines are capable of altering central 5-HT neurotransmission. For example, endotoxin LPS administration activated catecholamine metabolism and increased tryptophan in the brain of mice and rats (Mefford and Heyes, 1990; Dunn, 1992; Linthorst et al., 1995). Furthermore, peripheral IL-1β administration increased levels of 5-HT or its metabolite 5-HTIAA in several brain areas and increased 5-HT_1B_ and 5-HT_2C_ receptor expression in the hippocampus (Gemma et al., 1997; Connor et al., 1998; Dunn, 2006; Anisman et al., 2008), whereas IL-1 receptor antagonists reduced extracellular 5-HT in the hypothalamus (El-Haj et al., 2002). Conversely, in the periphery, 5-HT has immunoregulatory functions via its actions on 5-HT receptors expressed on immune cells (Mössner and Lesch, 1998; Herr et al., 2017). It has been shown that antidepressants, such as imipramine and fluoxetine, have anti-inflammatory effects and reduce the production of the pro-inflammatory cytokine IFN-γ while increasing production of the anti-inflammatory cytokine IL-10 (Maes et al., 1999; Kubera et al., 2001; Ramirez et al., 2015; Köhler et al., 2017). However, these findings need to be interpreted with caution, given that other studies have reported that SSRI treatment increases several pro-inflammatory cytokines in the brain, and that this elevation is necessary to produce the anti-depressant effects of SSRIs (Warner-Schmidt et al., 2011).

In most species, the expression of aggression is generally not pathological but adaptive. Therefore, the link between immune function, aggression, and depression that is observed in humans cannot be simply studied in ethologically relevant animal models of aggression. Rather, it will be appropriate for future studies to utilize animal models of escalated or pathological aggression when investigating the specific role of the immune system in aggression that is relevant to human psychiatric disease. For example, social isolation stress has been shown to induce escalated aggression as well as depressive-like behaviors in rodents (Haller et al., 2014). There are other animal models of escalated aggression considered to be relevant to the study of human aggression (Miczek et al., 2013). These animal models should be used in future studies aimed at understanding the immunobiology of aggression and its relation to depression.

## Conclusion and future perspectives

Findings from studies using animal models of social stress have uncovered numerous neurobiological mechanisms of immune—brain interactions that have opened up new avenues for anti-depressant drug discovery. In contrast, studies to define the neurobiological mechanism of immune—brain interactions in pathological aggression have lagged far behind, which some have argued reflects the relative paucity in novel drug targets for the treatment of psychiatric conditions with high aggression. Thus, we need a much better understanding of the neural circuits affected by the immune system involved in aggressive behavior. The use of animal models of escalated pathological aggression will help us understand relevant immunobiological mechanisms driving aggressive behavior and provide insight into the possible causes of pathological human aggression. Additionally, it is important to better define how the immune system interfaces with brain circuitry to control aggression. Future studies will have to consider whether individual differences in the immune system are causally linked with aggression, or whether the differences in immune function are simply a consequence of differing amounts of aggressive behaviors.

We currently have a very limited understanding of the neuro-immune interactions mediating aggression in females. In human studies, positive correlations between aggression and peripheral cytokine levels are observed in both males and females, yet rodent studies have not tested causal immunological mechanisms of aggression in females. Future studies will be required to clarify the degree of overlap in the immunological mechanisms that mediate male and female aggression. Given that that there are significant basal sex differences in the immune system (Klein and Flanagan, 2016), it is hypothesized that the immunological mechanisms driving aggression in males and females are different and highly dependent upon gonadal hormone status.

## Author contributions

AT, MF, BM, and SR discussed about the content of this review and wrote the manuscript. All authors read and approved the manuscript.

### Conflict of interest statement

The authors declare that the research was conducted in the absence of any commercial or financial relationships that could be construed as a potential conflict of interest.
